# Effects of a Phosphorus Flame Retardant System on the Mechanical and Fire Behavior of Microcellular ABS

**DOI:** 10.3390/polym11010030

**Published:** 2018-12-26

**Authors:** Vera Realinho, David Arencón, Marcelo Antunes, José Ignacio Velasco

**Affiliations:** Centre Català del Plàstic, Departament de Ciència dels Materials i Enginyeria Metal·lúrgica, Universitat Politècnica de Catalunya (UPC Barcelona Tech), C/Colom 114, E-08222 Terrassa, Barcelona, Spain; david.arencon@upc.edu (D.A.); marcelo.antunes@upc.edu (M.A.); jose.ignacio.velasco@upc.edu (J.I.V.)

**Keywords:** flame-retardant ABS microcellular foams, phosphorus flame retardants, MuCell^®^ injection-molding foaming

## Abstract

The present work deals with the study of phosphorus flame retardant microcellular acrylonitrile–butadiene–styrene (ABS) parts and the effects of weight reduction on the fire and mechanical performance. Phosphorus-based flame retardant additives (PFR), aluminum diethylphosphinate and ammonium polyphosphate, were used as a more environmentally friendly alternative to halogenated flame retardants. A 25 wt % of such PFR system was added to the polymer using a co-rotating twin-screw extruder. Subsequently, microcellular parts with 10, 15, and 20% of nominal weight reduction were prepared using a MuCell^®^ injection-molding process. The results indicate that the presence of PFR particles increased the storage modulus and decreased the impact energy determined by means of dynamic-mechanical-thermal analysis and falling weight impact tests respectively. Nevertheless, the reduction of impact energy was found to be lower in ABS/PFR samples than in neat ABS with increasing weight reduction. This effect was attributed to the lower cell sizes and higher cell densities of the microcellular core of ABS/PFR parts. All ABS/PFR foams showed a self-extinguishing behavior under UL-94 burning vertical tests, independently of the weight reduction. Gradual decreases of the second peak of heat release rate and time of combustion with similar intumescent effect were observed with increasing weight reduction under cone calorimeter tests.

## 1. Introduction

Acrylonitrile–butadiene–styrene (ABS) is one of the most used engineering polymers due its good combination of properties and low cost, being widely used in different industrial areas such as in the automotive sector, building, and construction, as well as in electrical and electronic applications. In the automobile sector, ABS is commonly used for interior and exterior car parts due to its high thermal insulation performance and the fact that its electrical properties do not change significantly with temperature and humidity [1]. Nevertheless, its high flammability with release of gases and toxic fumes during combustion significantly limits its use for this type of application.

Traditionally, the flame retardancy enhancement of polymeric materials such as plastics, foams, resins, and adhesives has been achieved through the use of brominated flame retardant additives. These materials were introduced in the 1960s and 1970s and are very effective at low concentrations [2]. However, the use of these halogenated flame retardants was demonstrated in the 1990s to adversely affect the environment due to high toxicity and bioaccumulation. In the past decade, the use of such additives has been highly limited due to European environmental restrictions [3], in some cases even resulting in their removal from the market as in the case of octabromodiphenyl oxide (OCTA) [4] and several brominated diphenyl ethers (BDEs) [5,6], making it critical to find alternative halogen-free flame retardant formulations.

Although styrenic polymers such as ABS are non-charring polymers, halogen-free phosphorous-based flame retardants (elemental red phosphorous, phosphines, phosphonium compounds, phosphonates, phosphites, phosphinates, and phosphates) are still the most used alternative to halogen-based FRs [7]. It is known that organophosphorus compounds in which phosphorus displays a high level of oxygenation (e.g., phosphates) decompose to form phosphorus acids that promote cationic crosslinking/char formation [8]. However, those containing phosphorus with a low level of oxygenation (e.g., phosphonates, phosphinates) generally decompose to liberate PO· radicals to the gas phase, where it scavenges combustion propagating radicals [9]. Phosphorus compounds with different mechanisms of action have been combined [10], as well as phosphorus and nitrogen compounds [11], to establish synergistic effects and enhance the fire performance. Moreover, expandable graphite or products derived from biomass [12], montmorillonite [13], layered double hydroxide [14], or carbon nanotubes [11,15] have been shown to act as synergist in phosphorus flame retardant systems.

Furthermore, there has been a great interest in some industrial sectors such as the automobile industry to replace conventional materials with lighter and eco-friendly alternatives. In this sense, due to its ease of processing and cellular structure control, styrenic-based foams, and particularly ABS foams, have experienced a great development, especially in terms of achieving microcellular or even nanocellular structures using foaming processes such as the MuCell^®^ injection-molding physical foaming process or the supercritical gas dissolution batch foaming process [16,17,18,19], hence achieving the best combination of weight reduction and mechanical performance.

Efforts to characterize the mechanical performance of polymer foams have been made during the past years, focusing on aspects such as strength and stiffness, energy absorption, impact strength, creep behavior, and dynamic-mechanical properties, as well as the influence of foam aspects such as composition, density, and cellular structure [20,21]. Particularly, several reports have considered the mechanical characterization of microcellular ABS-based foams, focusing on specific aspects such as the effects of processing and addition of secondary phases on foam density and cellular structure morphology and, as a consequence, on the mechanical properties of the resulting microcellular foams [22,23,24]. The addition of secondary phases, especially nanometric-sized particles, has been shown to favor cell nucleation during foaming, contributing to cell size reduction and cell density enhancement, which, together with their reinforcement of the polymer phase, results in foams with enhanced stiffness, strength, and improved storage modulus [25]. Nevertheless, the dynamic-mechanical analysis of microcellular ABS-based foams is still quite incipient, mainly due to the multiphase complex nature of these materials and the high complexity of such analysis.

A vast number of reports describing the enhancement of the fire behavior of polyurethane foams have appeared. The effects of adding phosphorus-based agents [26,27,28], intumescent compounds [29,30,31,32], inorganic or hybrid layered materials [33,34,35] and other bio-based flame retardants [36,37,38] have been discussed. However, the research and development of flame retardant styrenic foams [39,40,41,42] has received little attention. Even less has been reported for ABS foams. Consequently, the development of environmentally friendly ABS foams that meet demanding fire protection requirements remains a major challenge.

With all that in mind, the present work focuses on the study of the effects of weight reduction on the mechanical and fire performance of phosphorus flame retardant ABS structural microcellular parts prepared by MuCell^®^ injection-molding foaming. From this study, it was possible to note that weight reduction did not alter the self-extinguish behavior of the ABS flame retardant material. Unfoamed and foamed ABS/PFR parts showed a higher storage modulus than unfoamed ABS. Furthermore, the reduction of impact energy was smoother in ABS/PFR than in ABS parts with increasing weight reduction. Hence, coming as promising structural materials for fire proofing weight saving applications.

## 2. Materials and Methods 

An acrylonitrile–butadiene–styrene copolymer (ABS), with the commercial name ELIX^TM^ 128 IG, was provided by Elix Polymers (Tarragona, Spain). According to the manufacturer, ABS contains 26–28 wt % of butadiene in a matrix of styrene acrylonitrile (SAN) and has a melt volume rate of 15 cm^3^/10 min, measured at 220 °C and 10 kg. An ammonium polyphosphate (APP), Exolit^®^ AP422, and an aluminum diethylphosphinate (AlPi), Exolit^®^ OP1230, both supplied by Clariant Produkte (Sulzbach, Germany), were used as flame retardants. The APP, with chemical formula (NH_4_PO_3_)_n_, possesses a polymerization degree (n) higher than 1000 and a phosphorus and nitrogen content of 31–32 wt % and 14–15 wt % respectively, a density of 1.90 g/cm^3^ and an average particle size of 15 µm. The AlPi, with chemical formula [(C_2_H_5_)_2_PO_2_]_3_Al, has a phosphorus content of 23.3–24.0 wt %, a density of 1.35 g/cm^3^ and an average particle size of 30 µm, as reported by the manufacturer.

Before compounding, the ABS pellets and the phosphorus flame retardants (PFR) powders were respectively pre-conditioned at 80 °C during 4h and at 100 °C during 12h.

Neat ABS and ABS containing 12.5 wt % of APP and 12.5 wt % of AlPi (so-called for now on ABS/PFR) were melt-mixed in a co-rotating twin-screw extruder (Collin ZK-36, Germany) at a constant rotating speed of 110 rpm and a temperature profile from entrance to die of 160–170–185–190–190 °C. At the end, the extrudates were water-cooled and pelletized. Prior to injection-molding, extruded pellets of neat ABS and ABS/PFR were dried at 80 °C for 4 h. This APP and AlPi ratio was previously studied in ABS, where the mechanisms and mode of action of APP/AlPi were discussed and related to the enhancement of the fire performance of ABS [43].

**Figure 1:**
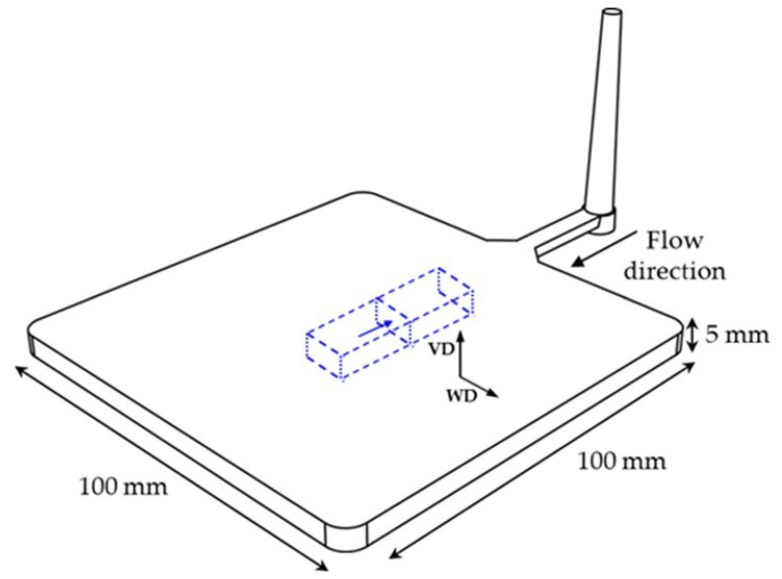
Scheme representing the square-shaped injection-molded part and the sample taken for dynamic-mechanical-thermal analysis (in blue). Blue arrow indicates the surface of the sample taken for the analysis of the cellular morphology of foams by scanning electron microscopy. VD: Vertical direction; WD: Width direction.

A Victory 110 injection-molding machine (Engel GmbH, Schwertberg, Austria) with a clamping force of 1100 kN, equipped with a 40 mm screw, a MuCell^®^ supercritical fluid (SCF) series II 25-mm injection valve, a SCF SII delivery system (Trexel Inc., Woburn, MA) for the conveying of SCF N_2_ and a mold temperature controlling device, were used for preparing the foamed parts. An injection temperature profile of 160–170–185–190–190 °C from hopper to nozzle was employed. The mold contained a 100 × 100 × 5 mm square-shaped plate cavity with a single fan gate located at one of the ends of the plate (see Figure 1). The N_2_ flow rate was kept constant at 0.25 kg/h, with dosage apertures of 2 s. A controlled mold temperature of 30 °C was used during a total time of 30 s. Melt plasticizing pressure was monitored at 19 MPa. Unfoamed parts were obtained as reference samples and foamed parts were injected with three different nominal weight reductions of 10%, 15%, and 20%. Foamed parts were identified as M-x, M being ABS or ABS/PFR and x the percentage of nominal weight reduction (10, 15, or 20). The experimental conditions were a result of a prior optimization of the injection-molding foaming process in order to obtain the mentioned nominal weight reductions.

The central zone of the parts was analyzed by means of scanning electron microscopy (SEM) using a JEOL JSM-5610 microscope. Samples were prepared by cryogenically fracturing the foams using liquid nitrogen and sputter depositing at their surfaces a thin layer of gold using a BAL-TEC SCD005 Sputter Coater. The values of the average cell size (*ϕ*), cell nucleation density (*N*_0_) and cell density (*N_f_*) of the core of all microcellular foams were determined from the analysis of a minimum of five characteristic ×500 magnification SEM micrographs taken from the foamed core according to the intercept counting method [44]. As can be seen in Figure 1, two cell sizes were determined according to the direction: *ϕ_VD_*, VD representing the vertical direction, i.e., the cell size in the thickness direction; and *ϕ_WD_* (WD—width direction). On the other hand, *N*_0_ and *N_f_* were calculated assuming an isotropic distribution of spherical cells according to Equations (1) and (2)

(1) $$N_{0}={\left(\frac{n}{A}\right)}^{3/2}\left(1-\rho_{rc}\right)$$

(2) $$N_{f}=\frac{6}{{\pi \phi }^{3}}\left(1-\rho_{rc}\right)$$

where in Equation (1) *n* is the number of cells in the micrograph, *A* (in cm^2^) its area, and *ρ_rc_* is the relative density, determined as the quotient between the density determined at the core center of the foamed parts and the density of the unfoamed reference material; and in Equation (2) *ϕ* is the average cell size determined as the average of the measured cell sizes in VD and WD directions (i.e., *ϕ*_VD_ and *ϕ*_WD_, respectively). In Equations (1) and (2), *N*_0_ represents the number of cells per volume of unfoamed material and *N_f_* the number of cells per volume of foamed material. Also, the relative density of the foamed parts, *ρ_r_*, was determined as the quotient between their density and the density of the unfoamed reference material.

Dynamic-mechanical-thermal analysis (DMTA) was used to study possible differences in the storage modulus, loss modulus and tan δ of the unfoamed and foamed parts. A DMA Q800 from TA Instruments (New Castle, DE, USA) was used and calibrated in a single cantilever configuration. The experiments were performed from −90 to 135 °C using liquid nitrogen at a constant heating rate of 2 °C/min and frequency of 1 Hz, applying a dynamic strain of 0.1%. Test specimens were cut from the center of the parts (see Figure 1) with a typical length of 30.0 ± 1.0 mm, width of 10.0 ± 1.0 mm, and thickness of 5.0 ± 0.1 mm.

Impact tests were carried out on an instrumented vertical falling weight testing machine CEAST Dartvis (Torino, Italy) at room temperature using a drop mass of 27.96 kg and a drop height of 1 m, according to ISO 6603-2 standard. The injection-molded parts (100 × 100 × 5 mm) were freely supported on a steel ring of inner diameter 40 mm and a transverse collision between the hemispherical indentation tip (20 mm diameter) and the part was applied. The impact force was recorded as a function of time by means of a piezoelectric force transducer with a load cell of 4 kN mounted on the head indentation tip. The signal was processed by the CEAST DAS 16000 advanced data acquisition system with a frequency of 1 MHz. Three different samples were tested for both unfoamed and foamed parts. Values of the maximum impact force (*F*_max_), energy absorbed until the maximum impact force (*E*_max_) and total absorbed energy (*E*_T_) were registered.

The flammability behavior was investigated using the UL-94 combustion vertical test on 125 × 13 × 5 mm specimens (cut directly from the injection-molded parts) ignited from bottom in the vertical configuration according to UL-94 standard (Underwriters Laboratories, USA).

Reaction-to-fire tests were carried out by means of a cone calorimeter (INELTEC, Barcelona, Spain) according to ISO 5660 standard procedure. Unfoamed and foamed specimens of 100 × 100 × 5 mm were irradiated with a constant heat flux of 50 kW/m^2^ using a constant distance between the electrical resistance and the specimen of 25 mm. Heat release rate (HRR) vs. time curves were registered during the tests. Typical fire-reaction parameters such as time to ignition (TTI), peak of the heat release rate (PHRR) and total heat emitted (THE) were obtained from the cone calorimeter tests.

## 3. Results

### 3.1. Structure of the Microcellular Parts

**Figure 2:**
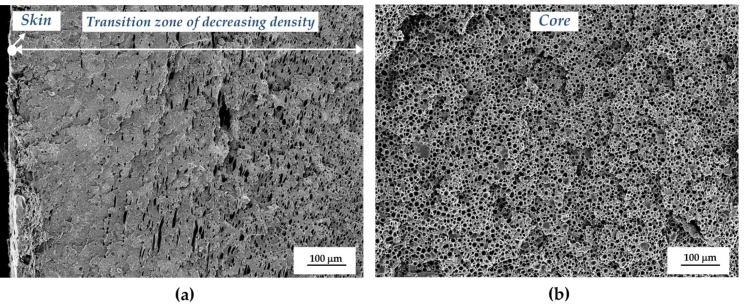
Low magnification SEM images showing the characteristic structure of injection-molded foams (ABS/PFR-15): (**a**) Skin and transition zone; (**b**) Core zone.

First of all, as can be seen by the characteristic low magnification SEM images displayed in Figure 2, the structural foams obtained by means of Mucell^®^ injection-molding foaming process showed a characteristic solid skin, a transition zone with decreasing density and a microcellular core structure. In proportion, the skins and transition zones represented around 30–40% of the whole thickness of the part and the microcellular core around 60–70%.

**Figure 3:**
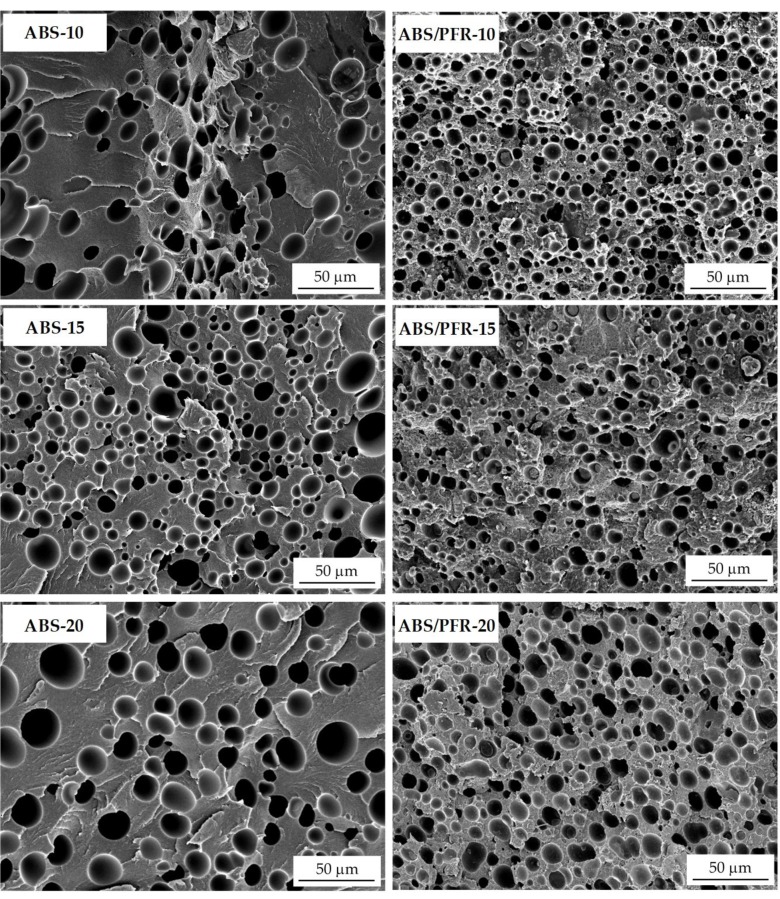
SEM images showing the characteristic core cellular morphology of ABS and ABS/PFR foams.

As can be seen from SEM images (Figure 3), clear differences could be observed between ABS and ABS/PFR’s core cellular structures. Particularly, ABS foams presented average cell sizes in both VD and WD directions higher than ABS/PFR foams, which were related to a heterogeneous cell nucleation effect promoted by the presence of the PFR particles (see Table 1). As a consequence, ABS/PFR foams displayed higher cell density and cell nucleation density values, reaching *N_f_* and *N*_0_ values clearly higher than 10^8^ cells/cm^3^, in one case even surpassing 10^9^ cells/cm^3^ (ABS/PFR-15). No clear relation was found between the weight reduction and the average cell size. For instance, ABS-15 foams presented lower average cell sizes than ABS-10 and ABS-20 foams, while ABS/PFR foams presented average cell sizes almost identical independently of the weight reduction (around 8–9 µm). Also, in terms of morphology all foams presented a homogeneous isotropic-like microcellular structure formed by spherical cells having aspect ratios (*ϕ*_VD_/*ϕ*_WD_) around 1.

**Figure 4:**
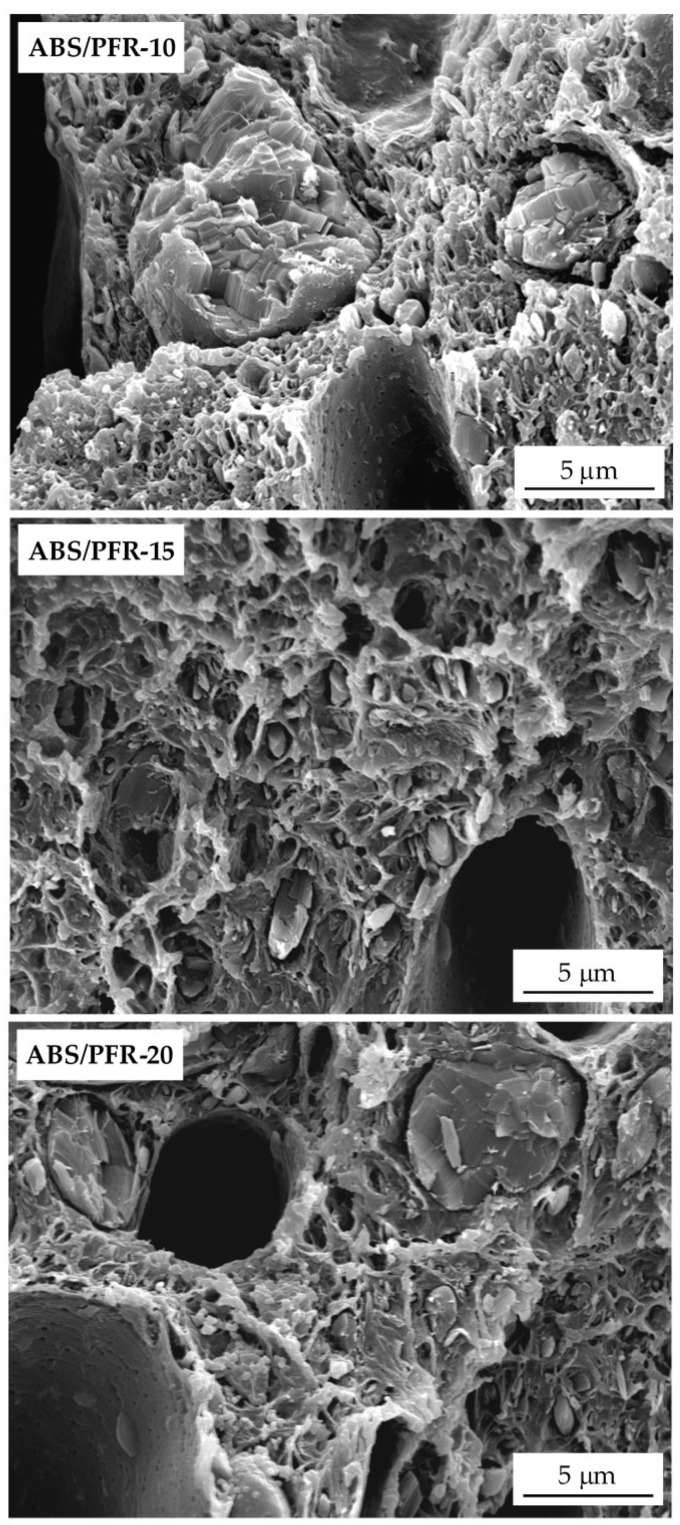
SEM images showing PFR particles throughout the cell walls of ABS/PFR foams.

In terms of PFR particles distribution, it can be seen by observing the characteristic SEM images presented in Figure 4 that, although the presence of particles having very different sizes, PFR particles were uniformly distributed throughout the cell walls of ABS/PFR foams.

### 3.2. Dynamic-Mechanical-Thermal Behavior

The storage modulus (*E*′), loss modulus (*E*″) and tan δ were obtained from DMTA. *E*′ indicates the ability of a material to storage elastic deformation energy, while *E*″ describes the energy dissipation of a material when it is deformed, being a measurement of the energy loss [45,46]; and tan δ gives a measure of the viscous fraction to the elastic one (tan δ = *E*″/*E*′). In the present work, the glass transition temperature (*T*_g_) of the rubbery and rigid phases were determined using tan δ curves. It should be mentioned that there was a slight difference between the onset and the end of tan δ associated to the glass transition of SAN. The end of the transition was taken as reference to determine its intensity (peak of tan δ—end of tan δ).

**Figure 5:**
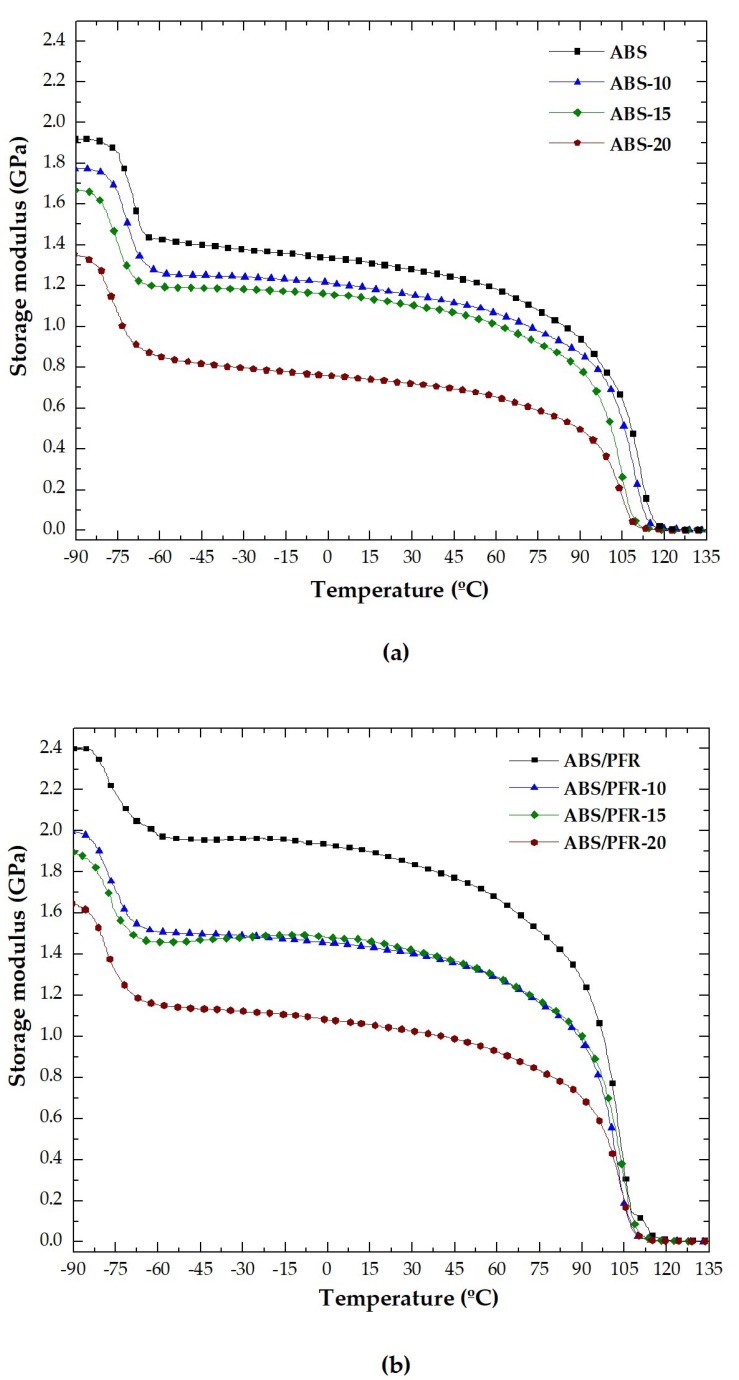
Evolution of the storage modulus with temperature for unfoamed and foamed (**a**) ABS and (**b**) ABS/PFR.

Figure 5a,b show the variation of the storage modulus with temperature. From these plots it was observed that there was a decrement in the value of the storage modulus at a temperature near −75 °C in the case of ABS and near −80 °C in the case of ABS/PFR. This phenomenon was related to a higher degree of free movement of some butadiene segments. At lower temperatures, the molecules of the glassy material have lower kinetic energies and their oscillations regarding their mean position are small, hence the materials presenting higher storage modulus values [47]. As the temperature increased, the storage modulus showed a sharp drop and then slowly decreased in the temperature region from −60 to 60 °C until reaching the energy of free movement of the SAN chain segments. For foamed samples, the beginning of the *E*′ decrement shifted towards lower temperatures.

**Figure 6:**
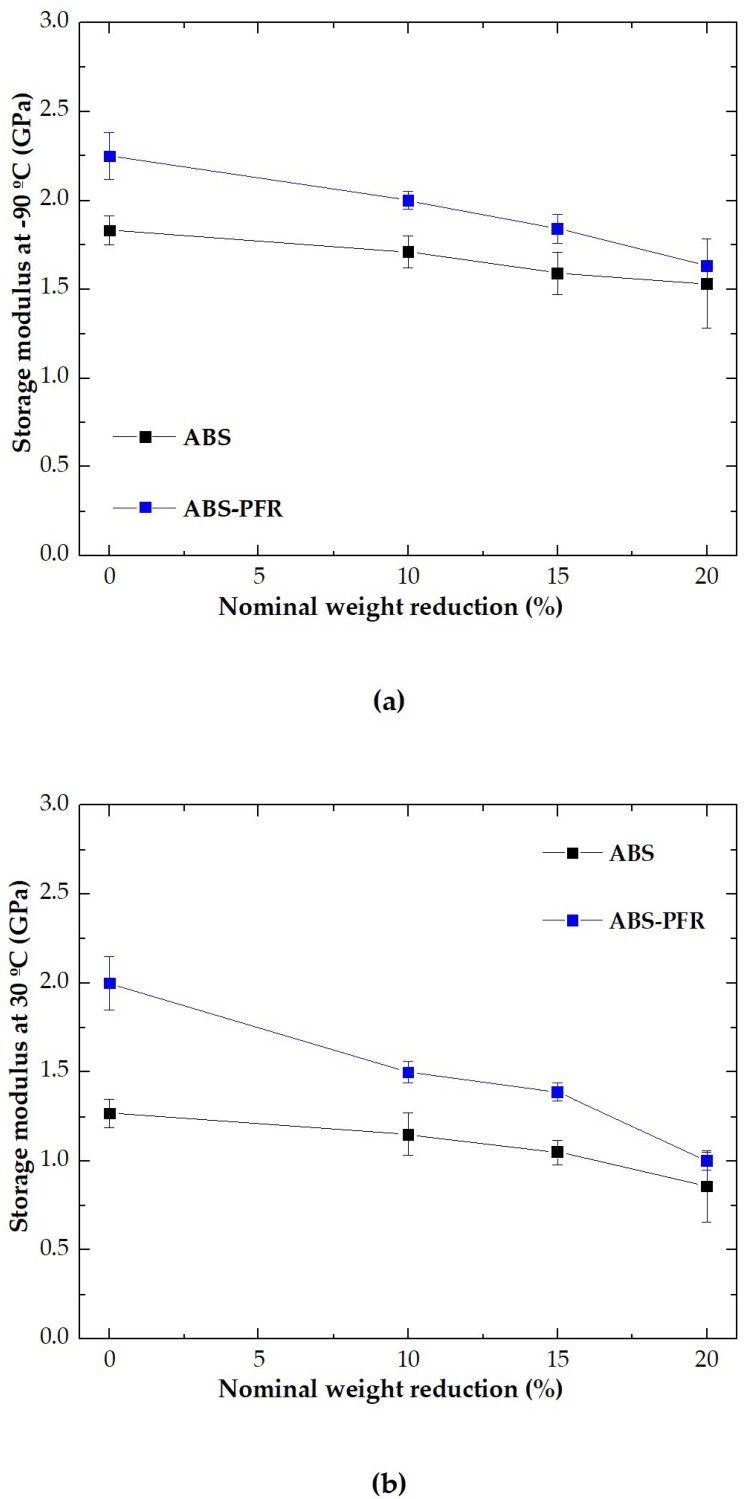
Variation of the storage modulus of ABS and ABS/PFR with the nominal weight reduction at (**a**) −90 °C and (**b**) 30 °C.

The values of *E*′ at −90 °C and 30 °C for the ABS and ABS/PFR materials versus nominal weight reduction are shown in Figure 6a,b, respectively. It was observed that the addition of the PFR system resulted in an enhancement of *E*′, increasing ABS’s energy storage capacity. Moreover, the value of this parameter decreased in both materials (ABS and ABS/PFR) as the nominal weight reduction increased. This fact indicates that the stiffness of ABS and ABS/PFR is affected by the foamed structure. However, ABS/PFR-20 foam showed an identical storage modulus to that of the unfoamed ABS, and ABS/PFR-10 and ABS/PFR-15 foams even higher.

**Figure 7:**
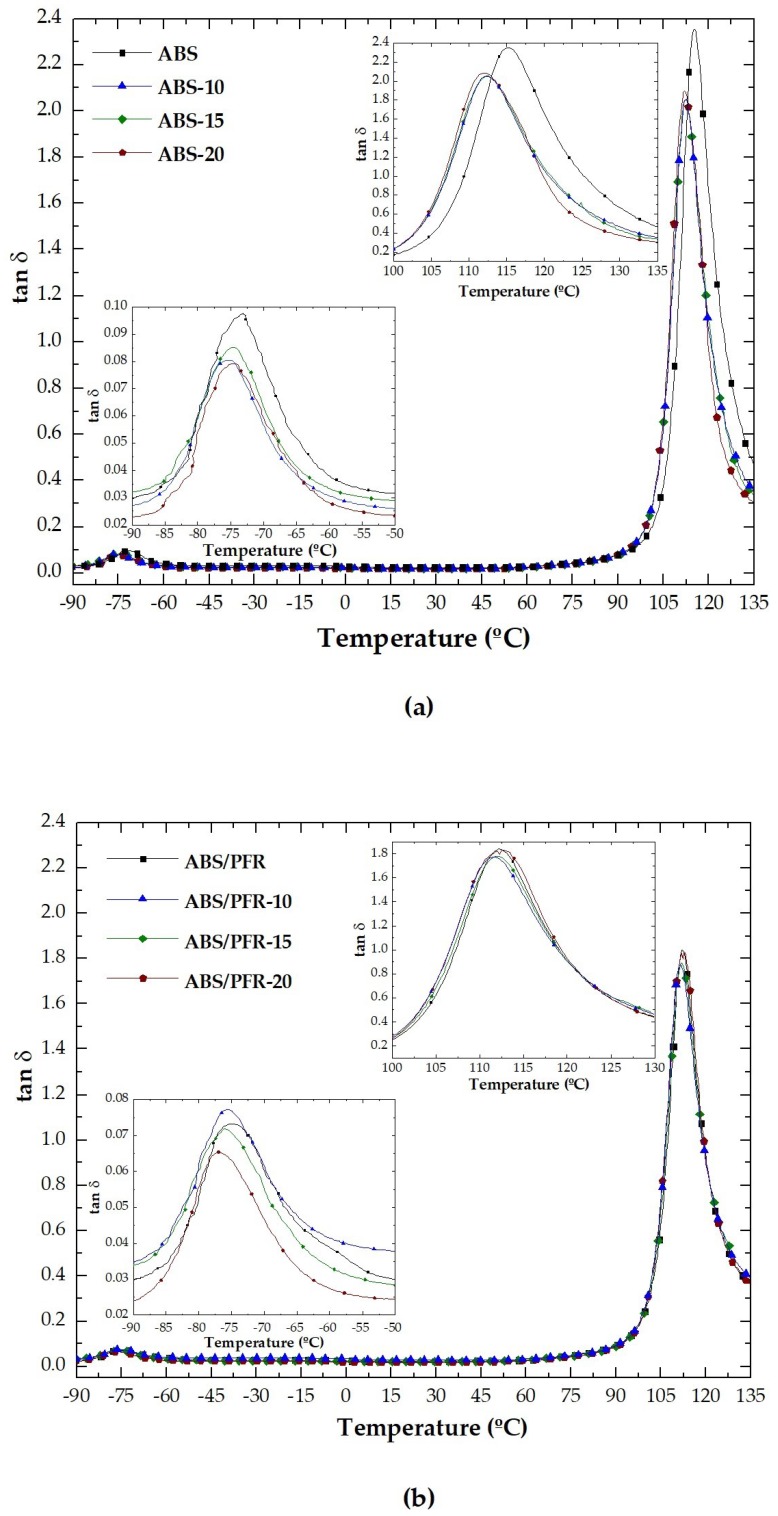
Evolution of the tan δ with temperature for the unfoamed and foamed (**a**) ABS and (**b**) ABS/PFR.

Figure 7a,b show the variation of the tan δ with temperature for unfoamed and foamed ABS and ABS/PFR. Two well-defined peaks corresponding to the relaxations associated to the glass transition of the rubbery and rigid phases were observed. The characteristic temperature corresponding to the peak point observed at lower temperature was related to the glass transition temperature of butadiene (*T*_g1_) and that observed at higher temperature to the styrene-acrylonitrile (SAN) (*T*_g2_) one [48].

**Figure 8:**
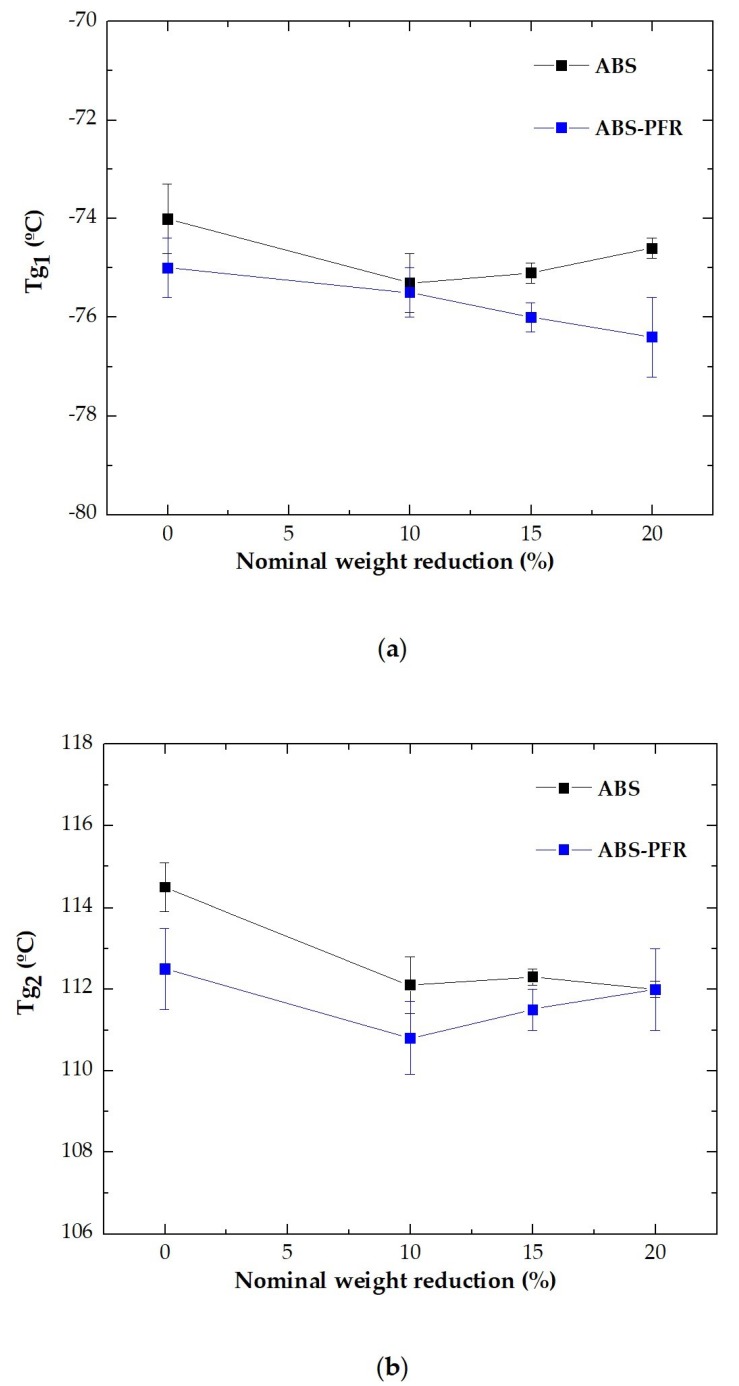
Variation of the glass transition of the (**a**) rubbery (*T*_g1_) and (**b**) rigid (*T*_g2_) phases of ABS and ABS/PFR with the nominal weight reduction.

Also, from Figure 8a,b it is possible to see with a greater detail that the incorporation of the PFR system slightly decreased the two glass transition temperatures of ABS (*T*_g1_ and *T*_g2_) and that the foamed parts globally displayed slightly lower values when compared to the respective unfoamed counterparts, indicating that the presence of the PFR particles and/or the microcellular structure of the foamed parts contributed to slightly decrease the chemical interactions between ABS’ macromolecules. The highest observed reduction was of 3.7 °C between the *T*_g2_ of the unfoamed ABS and ABS/PFR-10 foam.

**Figure 9:**
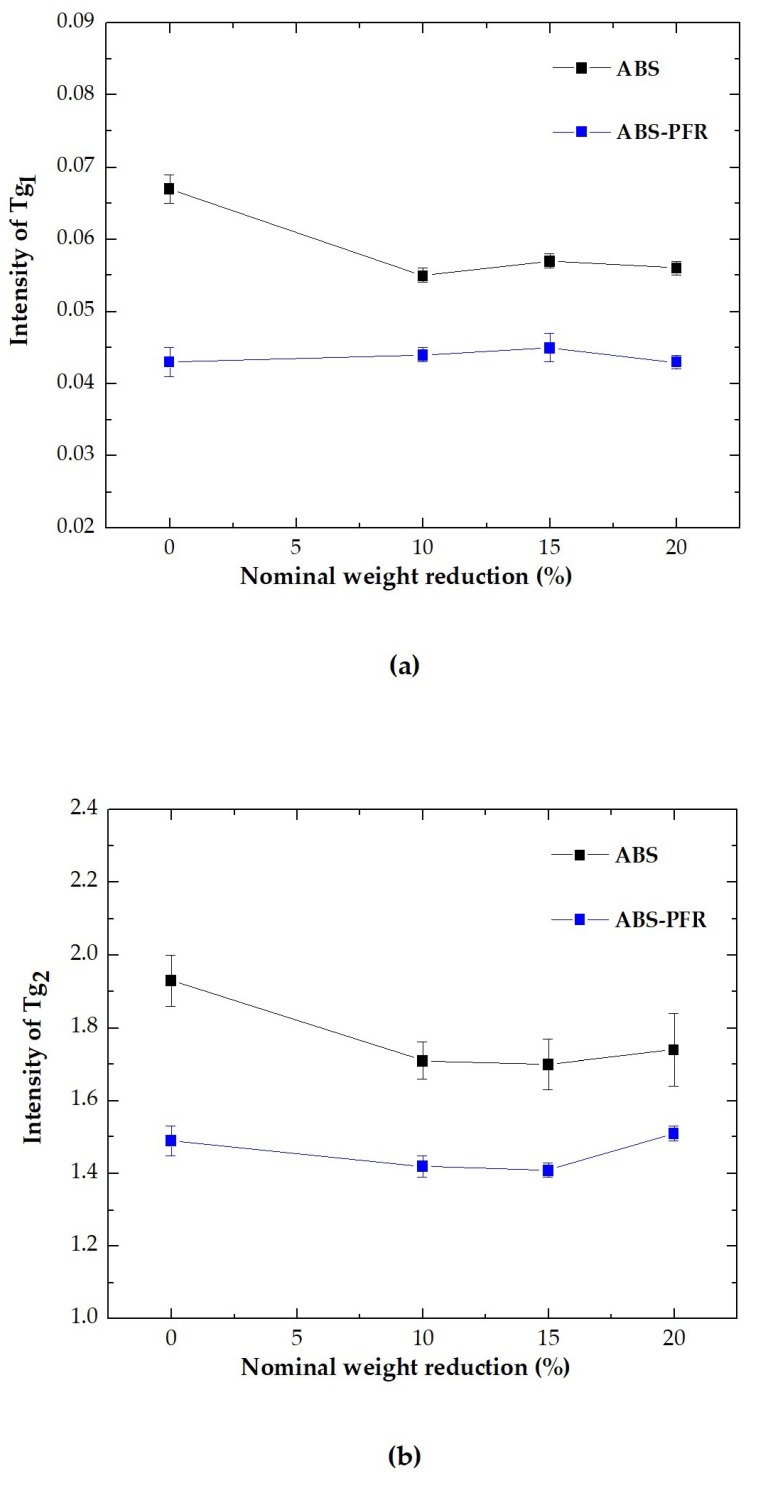
Variation of the intensity of tan δ of the (**a**) rubbery (*T*_g1_) and (**b**) rigid (*T*_g2_) phases of ABS and ABS/PFR with the nominal weight reduction.

Moreover, a chain mobility reduction was observed during the glass transitions of the rubbery and rigid phases with the presence of APP and AlPi particles (Figure 9a,b), resulting in a decrease in the damping properties. No influence of the weight reduction was noted on the intensity of the glass transitions of ABS/PFR, which indicates that the foamed structure had no influence on the molecular mobility of ABS when such particles were present.

### 3.3. Fracture Behavior

**Figure 10:**
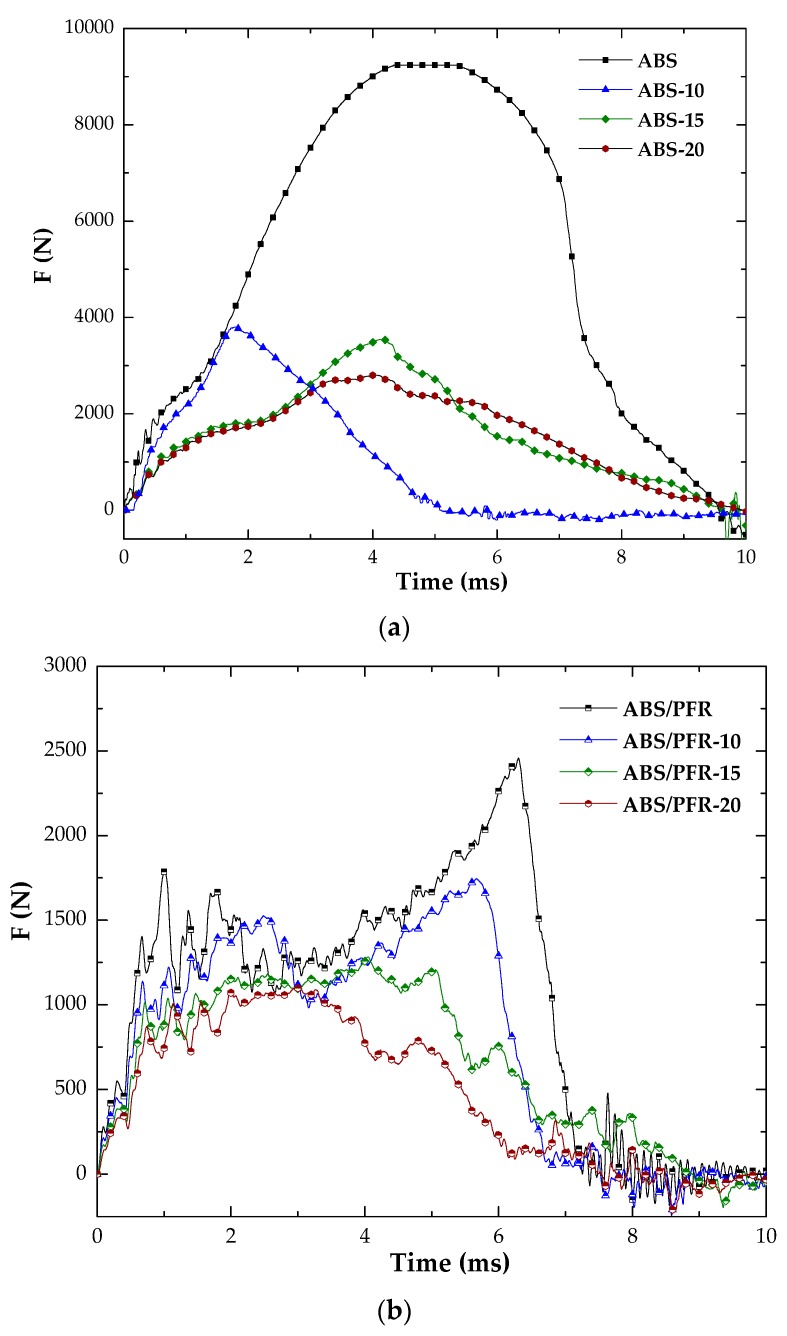
Characteristic force versus time falling weight curve of unfoamed and foamed (**a**) ABS and (**b**) ABS/PFR.

**Figure 11:**
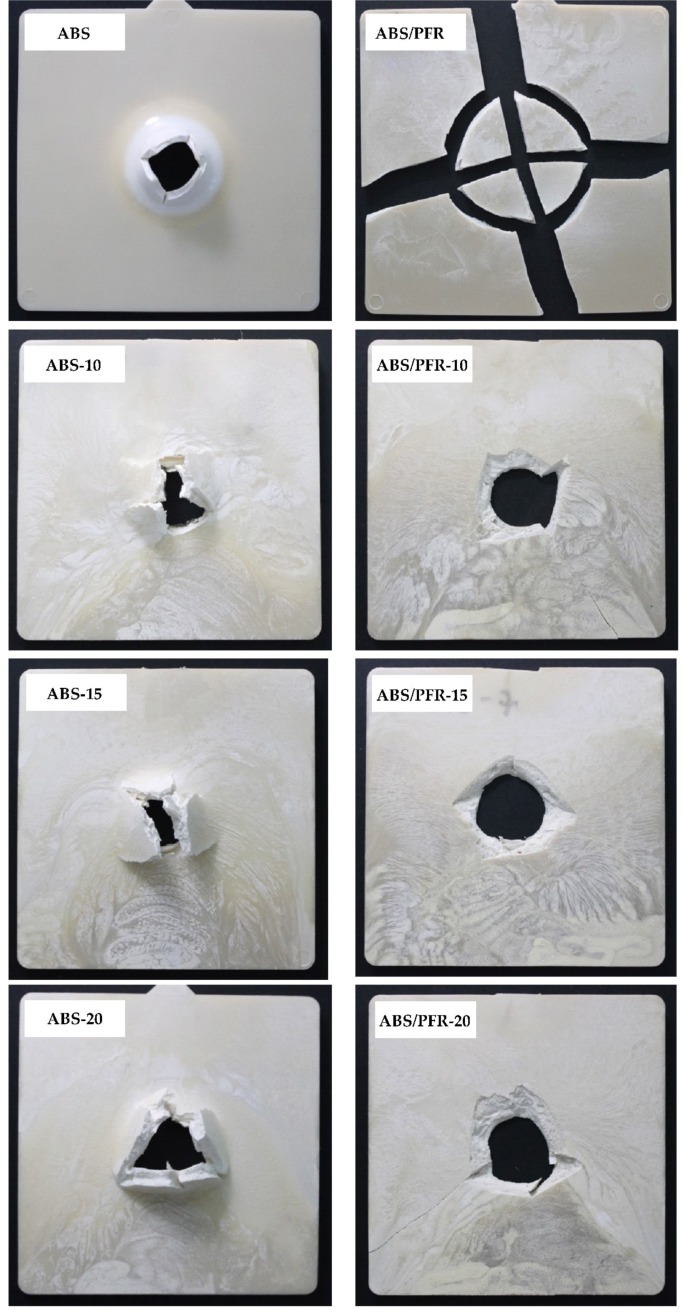
Characteristic images of unfoamed and foamed ABS and ABS/PFR parts after impact testing.

Figure 10 shows the characteristic falling weight impact curves and Figure 11 the parts after impact testing. From these figures it was possible to observe that unfoamed and foamed ABS parts displayed a ductile fracture pattern. Nevertheless, this ductile fracture pattern turned gradually to brittle with increasing weight reduction. Although the addition of the flame-retardant system led to a change in the fracture behavior from ductile to brittle with material detachment, the microcellular core structure developed in ABS/PFR specimens contributed in some way to minimize the brittle fracture pattern. This can be attributed to the role of cellular morphology, acting as a barrier against crack propagation [49].

Unfoamed parts displayed higher values of *F*_max_ than ABS and ABS/PFR foamed parts, as well as higher *E*_max_ and *E*_T_. As can be seen in Table 2, parts with a ductile behavior (without PFR) showed a significant difference between *E*_T_ and *E*_max_, much higher than in the case of ABS/PFR materials, which was related to a more significant plastic deformation during impact. Researchers have argued that the impact resistance reduction in cellular parts is due to the stress concentrator role of cells [50]. This implies that in materials with multiple crazing as the main mechanism of plastic deformation, the fracture behavior is brittle. This feature can also be increased by the presence of defects in the microcellular structure, such as big size bubbles or non-spherical cell geometries [51].

ABS/PFR showed lower *F*_max_ than pure ABS, as well as a totally brittle fracture. This behavior has also been observed in polymeric compounds with phosphorous-flame retardant additives [52]. A poor adhesion between additive and polymeric matrix is usually the main cause of this behavior. Nevertheless, comparatively the reduction in *F*_max_, *E*_max_, and *E*_T_ (see Table 2) was smoother in ABS/PFR than in ABS with increasing weight reduction. This effect is attributed to the lower cell size and higher cell density of ABS/PFR foams (see Figure 3). Quantitative studies have been performed on the influence of cell size and cell density on the fracture resistance of several amorphous and semicrystalline polymers, demonstrating that lower cell sizes and higher cell densities increase the fracture resistance, which was related to an increase of the surface area inside the material, acting as a barrier to crack propagation [49].

### 3.4. Fire Behavior

The flammability of both unfoamed and foamed parts was assessed by UL-94 vertical burning tests. From those, it was observed that the microcellular core structure of ABS or ABS/PFR foams did not change the materials’ behavior. Independently of the weight reduction, ABS burned completely and resulted in flammable drips that ignited the cotton (no rating in UL-94), while all ABS/PFR samples showed a self-extinguishing behavior (UL-94 V0 classification). This self-extinguishing behavior was associated to the flame inhibition promoted by the liberation of phosphorus radicals during the hydrolysis of AlPi and the formation of an effective protective layer due to the strong interactions between PFR particles and the polymer, being APP the major char-promoting component [43].

**Figure 12:**
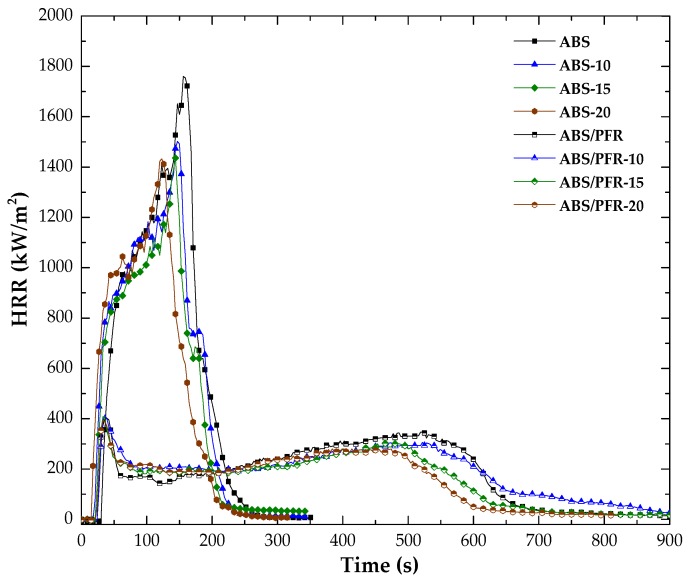
Characteristic HRR versus time curves of unfoamed and foamed ABS and ABS/PFR.

The forced flaming fire behavior was assessed by means of cone calorimeter tests. Figure 12 presents the characteristic heat release rate curves from said tests and Table 3 summarizes the main results.

As can be seen, the burning behavior of ABS and ABS/PFR gave rise to different characteristic HRR versus time curves. ABS showed a typical curve of a thermal intermediate thick non-charring and no residue forming material (as can be seen in Table 3). After ignition, the HRR values strongly increased until reaching a shoulder followed by a second increase of the HRR until reaching the maximum value (PHRR_1_). On the contrary, ABS/PFR showed a typical curve of a thermal thick charring material with an additional peak at the end of burning [53]. These curves showed an initial increase in HRR (until an efficient char layer was formed), followed by a quasi-static HRR plateau, with an average HRR value almost 5 times lower than that of unfoamed ABS. Although the existence of a second peak indicates a not completely efficient protective mode of action on the condensed phase, it should be noted that it occurred at a combustion time higher than 8 min without surpassing the value of the first peak. This highly efficient flame retardant effect of the PFR system was attributed to a combined gas and condensed-phase mode of action of the APP/AlPi in the ABS [34]. 

In a general way, by increasing the weight reduction of ABS and ABS/PFR, a gradual reduction of the TTI, PHRR, THE, and time of combustion were registered (see Figure 12 and Table 3). This is not surprising, taking into account the lower weight fraction of polymer under the radiant heat flux [54].

**Figure 13:**
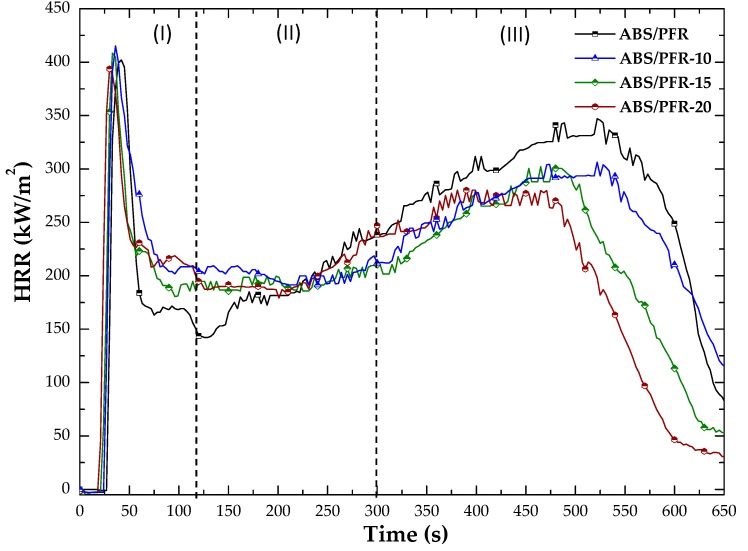
Characteristic HRR curves of unfoamed and foamed ABS/PFR parts showing the three main combustion stages.

Figure 13 shows the main stages of ABS/PFR heat release curves. After ignition (Stage I), no significant differences were observed between the PHRR_1_ of unfoamed and foamed ABS/PFR. This was related to a combined effect of the foamed samples solid skin and the PFR mode of action. In Stage II, the average value of the quasi-static HRR plateau value of foamed materials was slightly higher than the unfoamed one, being such difference related to the high surface area per unit mass of the core cellular structure of foams. However, in the last stage (Stage III), a gradual reduction of the second PHRR (PHRR_2_) and time of combustion (see Table 3) was observed as the weight reduction increased. This indicates that for higher times of combustion the low fuel contribution per unit volume of foamed ABS/PFR prevailed, contributing to a fire behavior enhancement. 

**Figure 14:**
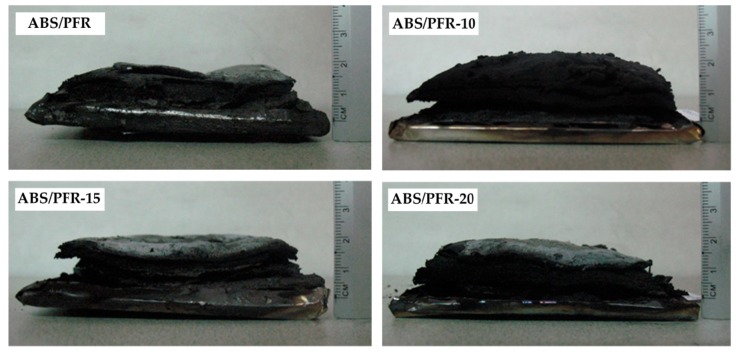
Residues of unfoamed and foamed ABS/PFR parts after the cone calorimeter tests.

Moreover, ABS/PFR foams showed similar residue contents (see Table 3) and residue expansion degrees (see Figure 14). This fact indicates that the weight reduction did not affect the intumescence of unfoamed ABS/PFR. Unfoamed and foamed ABS/PFR parts swelled and no collapse of the core structure was observed.

**Figure 15:**
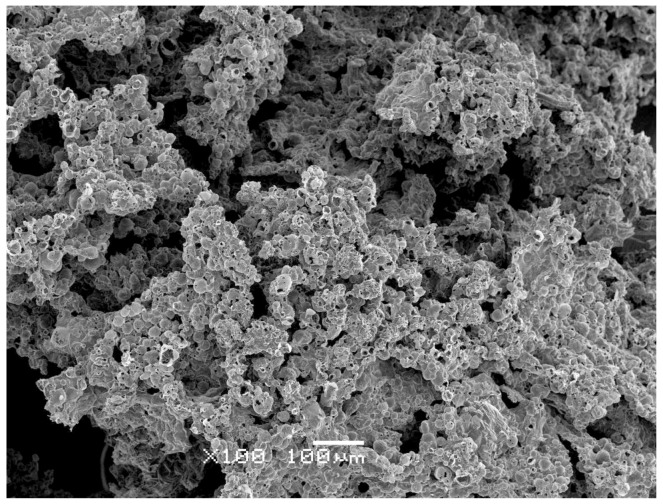
SEM micrograph showing the characteristic foamed morphology of a ABS/PFR char residue after cone calorimeter test.

The morphology of the char residue was also analyzed to assess the structure formed during combustion. All ABS/PFR parts, independently of the weight reduction, showed a porous carbonaceous structure (see Figure 15), which limited more effectively the heat and mass transfer from the flame to the underlying material. This condensed mode of action of the APP/AlPi system, combined with the releasing of its phosphorus radicals that worked like scavengers of the HO· and H· radicals yielded during ABS combustion [44], promoted a highly efficient flame retardant effect. This behavior is consistent with the self-extinguishing behavior observed during the UL-94 vertical burning tests of unfoamed and foamed ABS/PFR parts.

## 4. Conclusions

Microcellular ABS and ABS/PFR parts with 10, 15, and 20% of weight reduction were prepared and characterized. They presented a cellular structure gradient with skin, transition zone of decreasing density and a microcellular core. The addition of APP and AlPi into ABS promoted the formation of a more homogeneous microcellular core structure with smaller cell sizes and higher cell densities than neat ABS.

Both unfoamed and foamed ABS/PFR parts globally showed an increased storage modulus and a slight decrease of the butadiene and SAN glass transition temperatures compared to neat ABS. 

The incorporation of the PFR system and the foaming process led to a change in the ABS fracture behavior from ductile to brittle. Nevertheless, the reduction in impact energy was found to be lower in ABS/PFR parts than in neat ABS with increasing weight reduction.

Compared with the unfilled ABS, unfoamed and foamed ABS/PFR displayed an improved fire behavior. Both unfoamed and foamed ABS/PFR parts showed a UL-94 V0 classification and similar intumescent effect under the cone calorimeter tests. Compared with the unfoamed ABS/PFR, foamed ABS/PFR parts showed a gradual reduction of the second PHRR and time of combustion with increasing weight reduction.

## Figures and Tables

**Table 1:** Structural analysis of ABS and ABS/PFR microcellular foams

Material	Complete Part		Core					
	Density (g/cm^3^)	Relative Density, *ρ_r_*	Density (g/cm^3^)	Relative Density, *ρ_rc_*	*ϕ*_VD_ (µm)	*ϕ*_WD_ (µm)	*N_f_* (cells/cm^3^)	*N*_0_ (cells/cm^3^)
ABS-10	0.939	0.900	0.782	0.774	13.6	12.6	1.72 × 10^8^	1.35 × 10^8^
	(0.003)		(0.047)		(2.2)	(6.3)		
ABS-15	0.887	0.850	0.750	0.743	9.2	8.5	6.32 × 10^8^	6.46 × 10^8^
	(0.011)		(0.033)		(1.9)	(1.4)		
ABS-20	0.834	0.800	0.696	0.668	19.5	18.9	9.70 × 10^7^	8.07 × 10^7^
	(0.001)		(0.015)		(4.9)	(5.6)		
ABS/PFR-10	1.047	0.909	0.904	0.805	7.8	7.9	7.87 × 10^8^	6.88 × 10^8^
	(0.020)		(0.016)		(0.6)	(0.3)		
ABS/PFR-15	0.988	0.862	0.834	0.743	7.6	7.6	1.15 × 10^9^	1.60 × 10^9^
	(0.002)		(0.018)		(0.8)	(0.4)		
ABS/PFR-20	0.925	0.807	0.740	0.659	9.5	8.9	7.74 × 10^8^	9.68 × 10^8^
	(0.005)		(0.010)		(0.9)	(0.7)		

ABS density = 1.043 g/cm^3^; ABS/PFR density = 1.145 g/cm^3.^

**Table 2:** Falling weight impact results of unfoamed and foamed ABS and ABS/PFR parts

Material	*F*_max_ * (N)	*F*_max_ Reduction ** (%)	*E*_max_ * (J)	*E*_max_ Reduction ** (%)	*E*_T_ * (J)	*E*_T_ Reduction ** (%)
ABS	9385	-	79.9	-	119.6	-
ABS-10	3626	61.4	18.3	77.1	42.0	64.9
ABS-15	3314	64.7	16.6	79.2	28.9	75.8
ABS-20	2629	72.0	13.7	82.9	25.9	78.3
ABS/PFR	2439	-	9.5	-	10.7	-
ABS/PFR-10	1672	31.4	7.0	26.3	7.7	28.0
ABS/PFR-15	1297	46.8	5.3	44.2	6.6	38.3
ABS/PFR-20	1157	52.6	3.8	60.0	5.4	49.5

* Standard deviation values typically lower than 5%. ** Reduction of *F*_max_, *E*_max_, and *E*_T_ of microcellular parts over the reference unfoamed material.

**Table 3:** Results obtained from cone calorimeter tests.

Material	TTI (s)	PHRR_1_ * (kW/m^2^)	Time of PHRR_1_ (s)	PHRR_2_ * (kW/m^2^)	Time of PHRR_2_ (s)	THE (MJ/m^2^)	Residue (wt %)
ABS	32	1760	156	-	-	191	0.52
ABS-10	22	1502	147	-	-	178	0.50
ABS-15	22	1436	144	-	-	168	0.49
ABS-20	17	1432	123	-	-	157	0.47
ABS/PFR	28	402	39	345	522	173	11.3
ABS/PFR-10	28	415	36	306	522	162	11.3
ABS/PFR-15	25	409	33	301	483	140	13.0
ABS/PFR-20	23	409	33	280	468	132	12.6

* Standard deviation values typically lower than 2%.
